# Isolation and characterization of novel bacteriophages targeting *Stenotrophomonas maltophilia*

**DOI:** 10.1038/s41598-025-14811-5

**Published:** 2025-08-13

**Authors:** Wakana Yamashita, Yuta Sato, Matthew Imanaka, Michiyo Kataoka, Tadaki Suzuki, Aa Haeruman Azam, Shinjiro Ojima, Kayoko Hayakawa, Sho Saito, Yuki Moriyama, Norio Ohmagari, Masami Kurokawa, Kazuhisa Mezaki, Azumi Tamura, Longzhu Cui, Jumpei Fujiki, Hidetomo Iwano, Yoshimasa Takahashi, Koichi Watashi, Satoshi Tsuneda, Kotaro Kiga

**Affiliations:** 1 Research Center for Drug and Vaccine Development, National Institute of Infectious Diseases, Shinjuku-ku, Tokyo, 162-8640 Japan; 2 Department of Life Science and Medical Bioscience, Waseda University, Shinjuku-ku, Tokyo, 162-8480 Japan; 3 Department of Infectious Disease Pathology, National Institute of Infectious Diseases, Japan Institute for Health Security, Shinjuku-ku, Tokyo, 162-8640 Japan; 4 Department of Infectious Disease Pathobiology, Graduate school of Medicine, Chiba University, 260-8670 Chiba, Japan; 5 Disease Control and Prevention Center, National Center for Global Health and Medicine, Shinjuku-ku, Tokyo, 162-8655 Japan; 6 AMR Clinical Reference Center, Disease Control and Prevention Center, National Center for Global Health and Medicine, Tokyo, Japan; 7 Microbiology Laboratory, National Center for Global Health and Medicine, 1-21-1 Toyama, Shinjuku-ku, Tokyo, 162-8655 Japan; 8 Department of Infection and Immunity, School of Medicine, Jichi Medical University, Shimotsuke-shi, Tochigi 330-8503 Japan; 9 Department of Veterinary Medicine, Rakuno Gakuen University, Ebetsu-shi, Hokkaido 069-8501 Japan; 10 Phage Therapy Institute, Comprehensive Research Organization, Waseda University, Shinjuku-ku, Tokyo, 162-8480 Japan

**Keywords:** Bacteria, Bacteriophages

## Abstract

*Stenotrophomonas maltophilia* is a bacterium often resistant to antibiotics and is a significant cause of nosocomial infections, particularly in immunocompromised patients. Phage therapy has shown promise as a potential treatment for such difficult-to-treat bacterial infections, but research on phages targeting this bacterium is very limited. In this study, we isolated 34 phages using four clinical strains of *S. maltophilia* and evaluated their infectivity and bactericidal activity. While some phages infected all four strains, many exhibited strain-specific infectivity. We investigated the bacterial growth curves in response to three phages, named Yut1, Yut2, and Yut4, and found that all phages exhibited potent lytic activity against the clinical strains even at low doses. Genome analysis found that the phages did not carry any lysogeny genes, virulence factors, or antibiotic resistance genes, suggesting their high potential as therapeutic phages. Furthermore, phylogenetic analysis suggested that Yut1 and Yut4 belong to a novel phage lineage. These results highlight the therapeutic potential of our novel phages to combat the growing antibiotic resistance problem.

**Supplementary Information:**

The online version contains supplementary material available at 10.1038/s41598-025-14811-5.

## Introduction

*Stenotrophomonas maltophilia* is recognized as an opportunistic pathogen that poses a significant threat to immunocompromised individuals, particularly those undergoing immunosuppressive treatments or with a history of inflammatory lung diseases^1^. The mortality rate for infections caused by this bacterium can be as high as 37.3% among immunodeficient patients, underscoring the critical need for effective therapeutic interventions^2^.

*S. maltophilia* is also recognized for its multidrug resistance, which has become a major public health concern^3^. It has developed resistance to most classes of antibiotics, including β-lactams, cephalosporins, aminoglycosides, and macrolides, making treatment options increasingly limited^4^. Trimethoprim-sulfamethoxazole (TMP-SMX) is often used as the first-line treatment for *S. maltophilia* infections^5^. When TMP-SMX is ineffective, other antibiotics such as minocycline, levofloxacin and cefiderocol, or a combination of ceftazidime/avibactam with aztreonam, are employed as last-line options^6,7^. However, the emergence of *S. maltophilia* strains resistant to these drugs has become a significant concern^8^. While infections are most commonly associated with hospital settings, cases of community-acquired infections have also been reported, indicating a broader impact on public health^9,10^. Given the limited efficacy of conventional antibiotics, there is an urgent need to explore alternative treatment options^11^.

Despite the need for alternative treatments for *S. maltophilia*, research on phage therapy remains limited^12,13^. To date, no clinical trials have been conducted in humans, and even in vivo studies utilizing animal models, such as mice, are not yet well established for *S. maltophilia*. This can be attributed to the limited isolation and characterization of *S. maltophilia*-specific phages. Currently, only 31 phages targeting *S. maltophilia* are registered in the International Committee on Taxonomy of Viruses (ICTV) database^14–17^, underscoring the urgent need for expanded efforts in phage isolation and characterization to address this critical pathogen.

In this study, we isolated 34 phages using four clinical isolates of *S. maltophilia* and assessed their infectivity against these bacterial strains. Additionally, we sequenced the genomes of three phages and performed phylogenetic analysis.

## Result

### Isolation and host range characterization of phages infecting *Stenotrophomonas maltophilia*

**Fig. 1:**
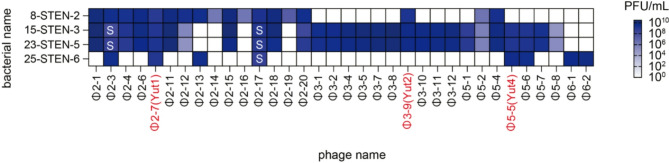
Heatmap of spot assay for 34 phages on *S. maltophilia.* A heatmap was generated based on the spot assay results of 34 isolated phages on the four *S. maltophilia* strains. The vertical axis represents the bacterial strains, and the horizontal axis represents the phage names. “S” indicates that the plaque size is smaller compared to the original host strain used for phage isolation.

Four *Stenotrophomonas maltophilia* strains (8-STEN-2, 15-STEN-3, 23-STEN-5, 25-STEN-6) were isolated from refractory patients in 2024. Among the patients from whom *S. maltophilia* was isolated, the patient with strain 8-STEN-2 had a mono-microbial infection, whereas the patients with the other three strains had polymicrobial infections. Additionally, various antibiotics were administered to these patients for treatment (Supplementary Table 1). Antimicrobial susceptibility testing of these strains revealed that 8-STEN-2 was sensitive to ceftazidime, minocycline, levofloxacin, and sulbactam/tazobactam, while strain 15-STEN-3 showed resistance to both ceftazidime and levofloxacin. 23-STEN-5 and 25-STEN-6 only exhibited ceftazidime resistance (Supplementary Table 2). Genomic analysis revealed that 15-STEN-3 and 23-STEN-5 shared highly similar genome sequences despite exhibiting different antibiotic susceptibility profiles, possibly due to differences in their plasmid content (Supplementary Fig. 1). Using these four isolates as hosts, phages were isolated from sewage collected in Tokyo. As a result, 15 phages were isolated using 8-STEN-2, 10 using 15-STEN-3, 7 using 23-STEN-5, and 2 using 25-STEN-6, leading to the successful isolation of a total of 34 *S. maltophilia* phages (Supplementary Table 3). The infectivity of these 34 phages was assessed using spot tests on the four clinical isolates. The results showed varying infectivity patterns among the phages (Fig. 1, Supplementary Fig. 2).

Notably, Φ2–7 was the only phage that produced large plaques against all clinical isolates. While Φ2–3 and Φ2–17 could also infect all four isolates, the plaque size varied depending on the strain. Φ2–3 formed small plaques on 15-STEN-3 and 23-STEN-5, and Φ2–17 formed small plaques on 15-STEN-3, 23-STEN-5, and 25-STEN-6 (Fig. 1, Supplementary Fig. 2). Based on these results, we decided to further investigate Φ2–7, which we considered the most promising candidate for phage therapy. Given that large plaques are generally associated with higher therapeutic potential, we decided to study phages with different properties from Φ2–7 in parallel. Φ3–9 and Φ5–5 were selected because they have clearly different host ranges from Φ2–7 and were isolated using different bacterial strains. Φ3–9 showed infectivity towards 8-STEN-2, 15-STEN-3, and 23-STEN-5, while Φ5–5 towards 15-STEN-3, 23-STEN-5, and 25-STEN-6. Φ2–7, Φ3–9, and Φ5–5 were named as Yut1, Yut2, and Yut4, respectively. We also gained insights into the phage susceptibility of the clinical isolates used in this study. Phages isolated against 15-STEN-3 were also effective against 23-STEN-5, and vice versa, likely reflecting the high genomic similarity between 15-STEN-3 and 23-STEN-5. In contrast, 25-STEN-6 showed relatively high resistance to phages isolated from other strains, and phage isolation against this strain was challenging. Nevertheless, we successfully isolated phages with strong infectivity against all four strains.

### Bactericidal activity of three *Stenotrophomonas maltophilia* phages in liquid culture

**Fig. 2:**
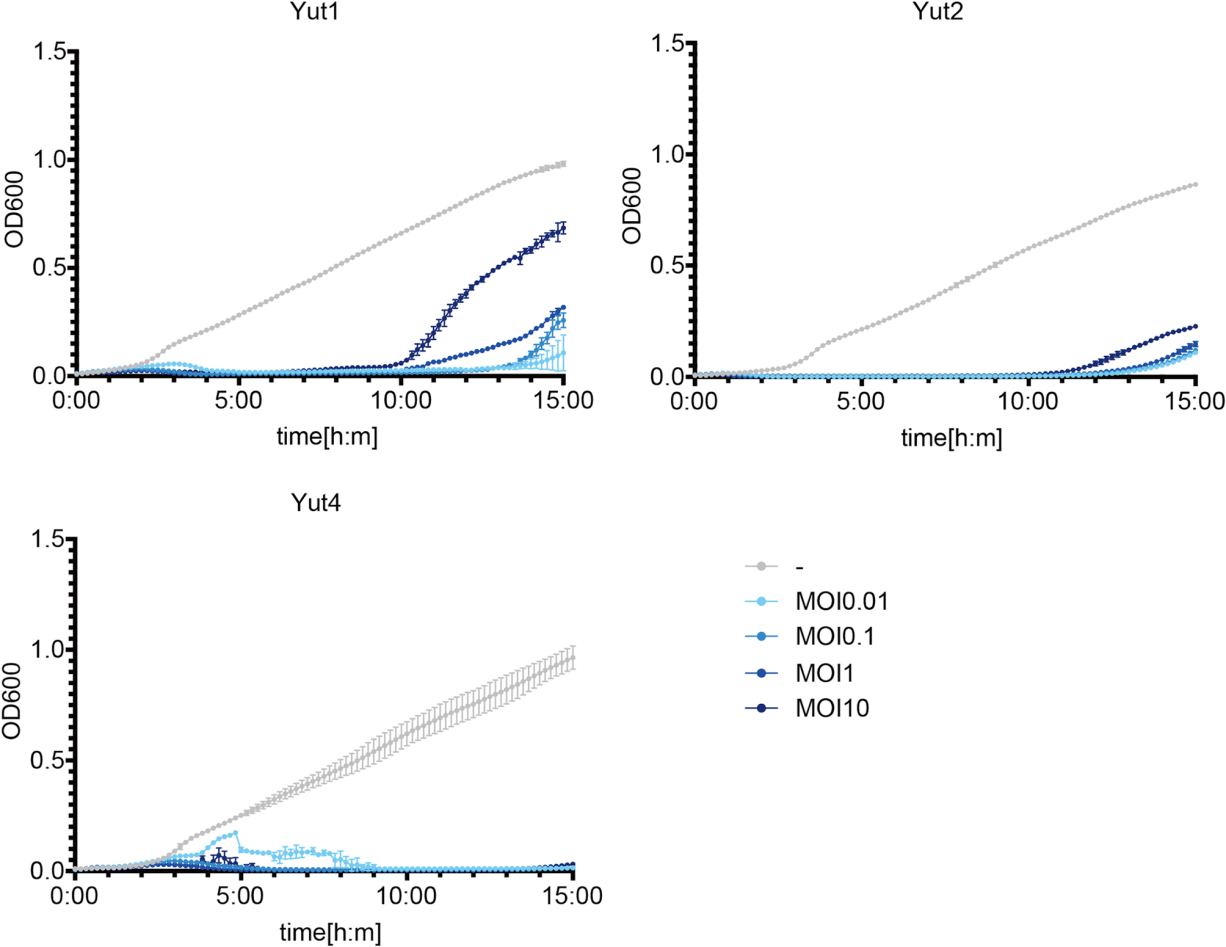
Bactericidal activity of isolated phages Yut1, Yut2 and Yut4. Line graphs depicting the bactericidal activity of phages Yut1, 2, 4 in liquid culture. Host bacteria (used for phage isolation) were inoculated with each phage at MOIs of 0.01, 0.1, 1, and 10, and bacterial growth curves were measured. Data represents the mean of three independent experiments (*n* = 3), with error bars indicating standard deviation.

From the host range test, three phages (Yut1, Yut2, and Yut4) with distinct host ranges were selected for further study. Each phage was inoculated to their respective isolation host at multiplicities of infection (MOI) of 0.01, 0.1, 1, and 10, followed by OD_600_ measurements. All phages demonstrated bactericidal activity in liquid culture, with higher MOIs resulting in more rapid bacterial clearance (Fig. 2).

Even at an MOI of 0.01, bactericidal activity was observed for all phages. Notably, Yut2 exhibited exceptionally strong bactericidal activity even at an MOI of 0.01, with almost no bacterial growth observed before the emergence of resistant bacteria. Similarly, Yut1 also showed stronger bactericidal activity compared to Yut4, consistently suppressing OD_600_ levels below 0.1 at an MOI of 0.01 prior to the emergence of resistant bacteria. In contrast to Yut1 and Yut2, there was slight bacterial growth for Yut4 at an MOI of 0.01. However, unlike Yut1 and Yut2, the emergence of resistant bacteria was not detected during the 15-hour period. In all phages, higher MOIs resulted in faster bactericidal activity but tended to accelerate the emergence of resistant strains.

To investigate the resistance mechanisms and identify potential phage receptors, we isolated three independent resistant mutants for each of the bacterial strains used for phage isolation: 8-STEN-2 for Yut1, 15-STEN-3 for Yut2, and 23-STEN-5 for Yut4 (Supplementary Fig. 3). Whole-genome sequencing of these mutants revealed common mutations in the fhuE receptor gene in all three 8-STEN-2 mutants resistant to Yut1, and in the colicin I receptor gene in all three 23-STEN-5 mutants resistant to Yut4 (Supplementary Table 4). Both the fhuE and colicin I receptor genes encode outer membrane proteins and play a role in the acquisition of ferric iron (Fe³⁺)^18,19^. In contrast, for 15-STEN-3 mutants resistant to Yut2, mutations were found in genes associated with pili function (twitching mobility protein) and cell wall biosynthesis (UDP-glucose 6-dehydrogenase, TuaD), but no mutations were commonly shared among all three resistant strains.

### Genomic characterization of three isolated *Stenotrophomonas* phages

The genomes of the three isolated phages, Yut1, Yut2, and Yut4, were sequenced and annotated. The genome sizes were determined to be 61,843 bp, 161,129 bp, and 62,174 bp, respectively (Table 1).

**Table 1:** Genomic data of Yut1, Yut2, and Yut4.

Phage	Genome size	ANI (The most similar phage)	GenBank acc. No.
Yut1	61,843 bp	93.51 (Salva)	PV130725
Yut2	161,129 bp	96.83 (IME SM1)	PV130723
Yut4	62,174 bp	92.55 (Salva)	PV130726

**Fig. 3:**
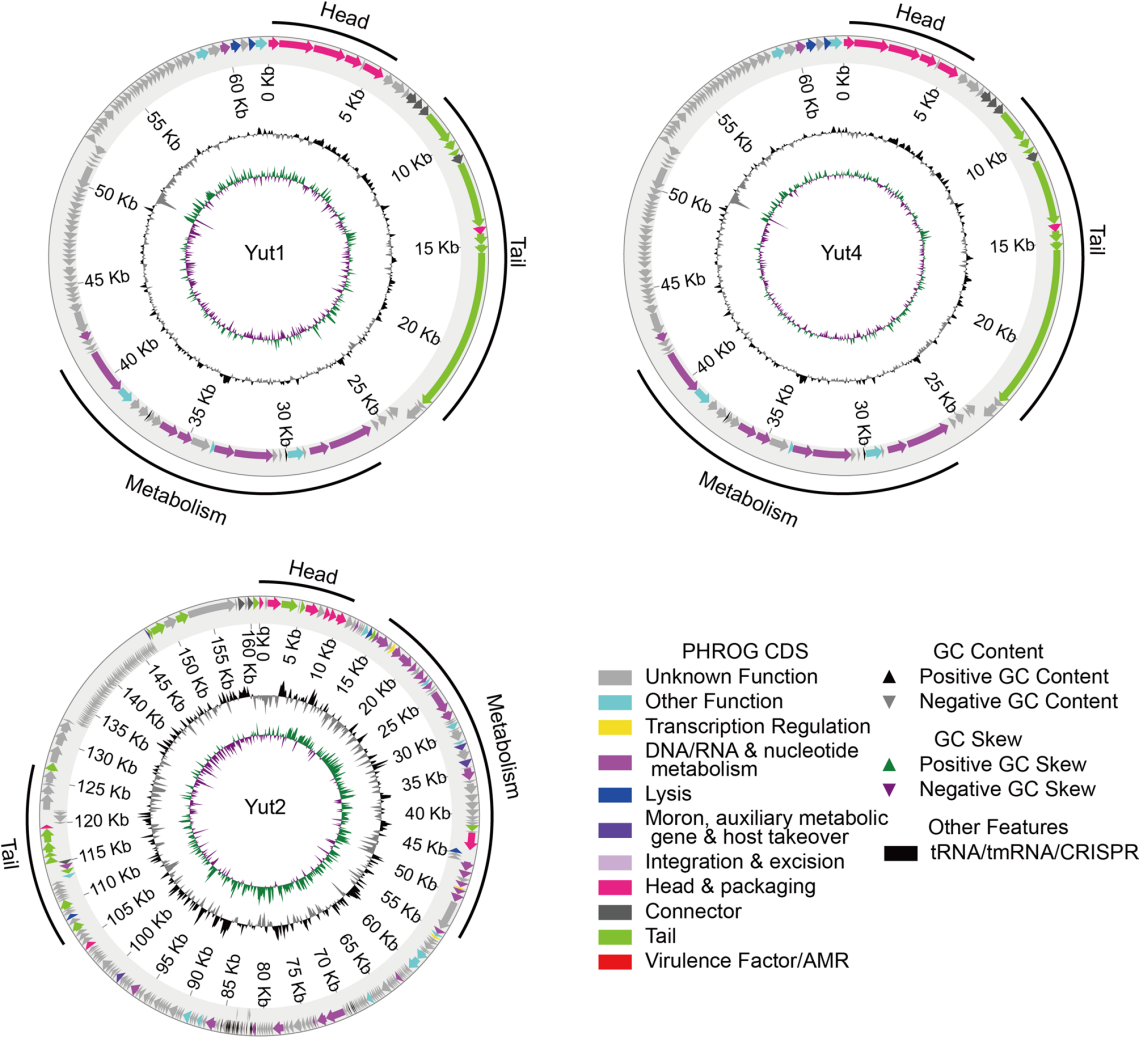
Genome maps of Yut1, Yut2, and Yut4. Circular genome maps of Yut1, Yut2, and Yut4 generated using Pharokka. The outermost circle represents predicted CDSs, with gene functional categories color-coded as indicated in the legend. The innermost circle shows the GC skew, while the middle circle represents the GC content.

The genome annotation results using Pharokka^20^ revealed that none of the phages encoded genes related to integration or excision, confirming their classification as lytic phages (Fig. 3).

Furthermore, no virulence factors and antimicrobial resistance (AMR) genes were detected (Fig. 3). In phage therapy, it is essential to use lytic phages that do not integrate into the genome of the target bacterial strain to avoid triggering unexpected bacterial evolution. Additionally, the use of phages that do not carry virulence factors and AMR genes is crucial to prevent the spread of resistance genes. We also investigated whether these phages possessed transduction capabilities. The phages were first used to infect 23-STEN-5, a gentamicin resistant strain (Supplementary Fig. 4). The resulting lysate was then used to infect 8-STEN-2 or 15-STEN-3, gentamicin susceptible strains. After phage infection, no gentamicin-resistant 8-STEN-2 or 15-STEN-3 colonies were observed, indicating that these phages do not mediate transduction (Supplementary Fig. 5). These findings highlight that the isolated lytic phages are promising candidates for phage therapy.

**Fig. 4:**
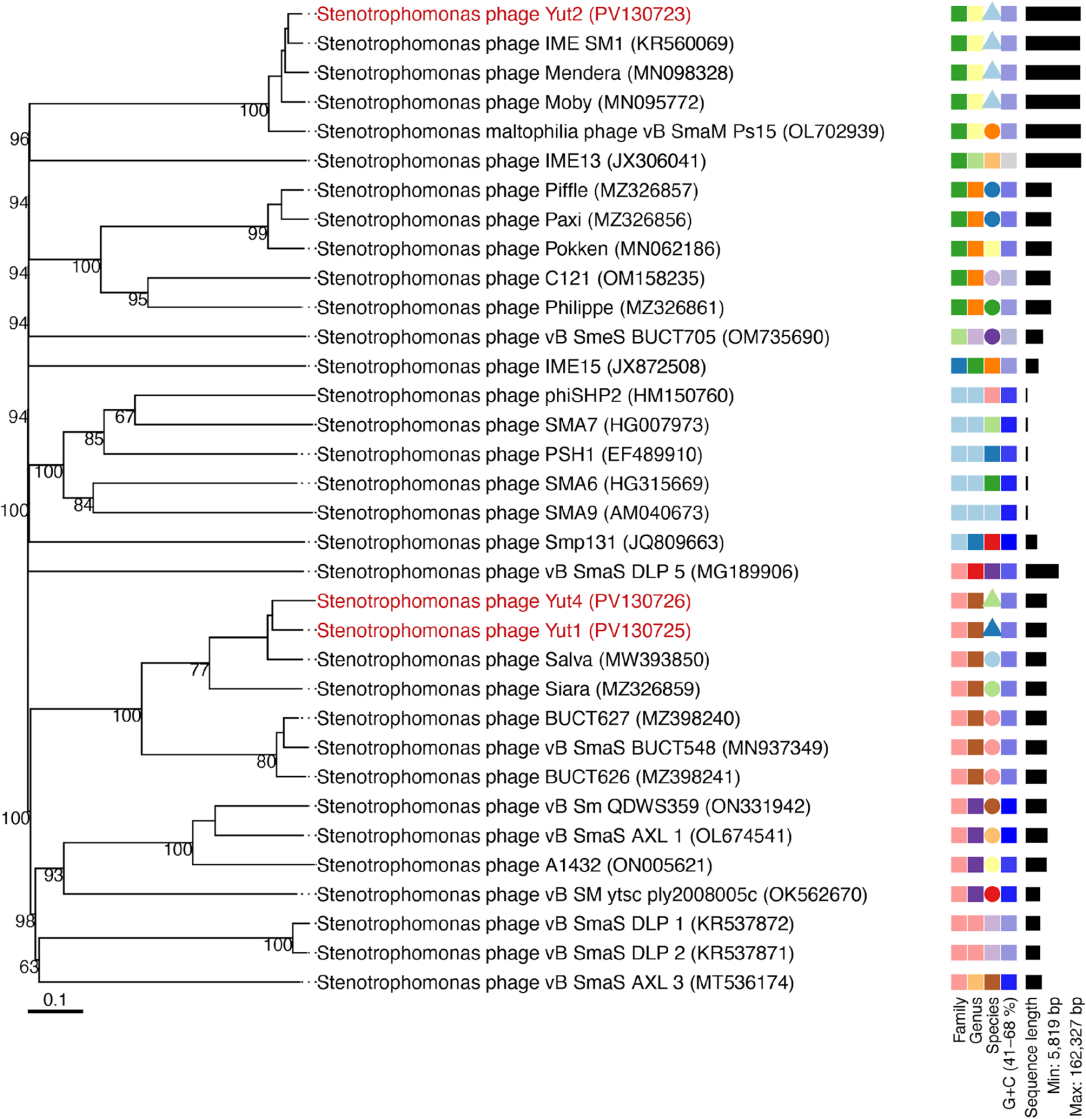
Phylogenetic analysis of Yut1, Yut2, and Yut4. Phylogenetic tree constructed using the VICTOR web tool^16,21^ based on whole-genome sequences of phages registered in the ICTV database and the three phages (Yut1, Yut2, and Yut4) isolated in this study. The phages isolated in this study are highlighted in red. Taxonomic classifications at the family, genus, and species levels, GC content, and genome length indicated by the bar graph are shown on the right for each phage. Taxonomic classifications were inferred based on calculated intergenomic distances.

Phylogenetic analysis was performed using nucleotide sequences of the four phages and all 31 *S. maltophilia* phages registered in the ICTV database. A phylogenetic tree constructed using VICTOR^16,21^. The analysis revealed high similarity between Yut1 and Yut4 with *Stenotrophomonas* phage Salva^22^ (Accession number: *NC_071017*), and between Yut2 with *Stenotrophomonas* phage IME SM1(Accession number: *NC_054952*) (Fig. 4).

Average nucleotide identity (ANI) was calculated for each phage to determine novelty. Yut1 exhibited an ANI of 93.51 compared to the closest related phage, Salva. Yut4 was also found to be related to Salva, with an ANI of 92.55. However, Yut1 and Yut4 share an ANI of 94.38. Following the ICTV guideline of 95, Yut1 and Yut4 are novel phages closely related to each other and Salva^23^. In contrast, Yut2 showed ANI values of 96.83 with *Stenotrophomonas* phage IME SM1, indicating a high degree of similarity to this previously characterized phage (Table 1).

Although Yut1 and Yut4 cluster closely in the phylogenetic tree, only Yut1 exhibited infectivity toward 8-STEN-2, whereas Yut4 was completely unable to infect this strain. Comparative genomic analysis revealed a high degree of similarity between the two phages (Supplementary Fig. 6). However, differences were observed in genes related to the tail structure, metabolism, and some hypothetical proteins with unknown functions (Supplementary Fig. 6). To further investigate the basis for their differing host ranges, we focused on the amino acid sequences of tail proteins, which are typically involved in host recognition. Annotation and alignment of the longest tail gene, central tail fiber protein J, using Clustal Omega (EMBL-EBI, Hinxton, UK)^24,25^ revealed a pairwise distance of 0.17434, indicating overall similarity with discernible divergence. Notably, the N-terminal region of the protein was almost identical between the two phages, while the C-terminal region showed sequence differences, suggesting that this region may be responsible for the observed difference in host specificity (Supplementary Fig. 7).

### Morphological characterization of phages using transmission electron microscopy

**Fig. 5:**
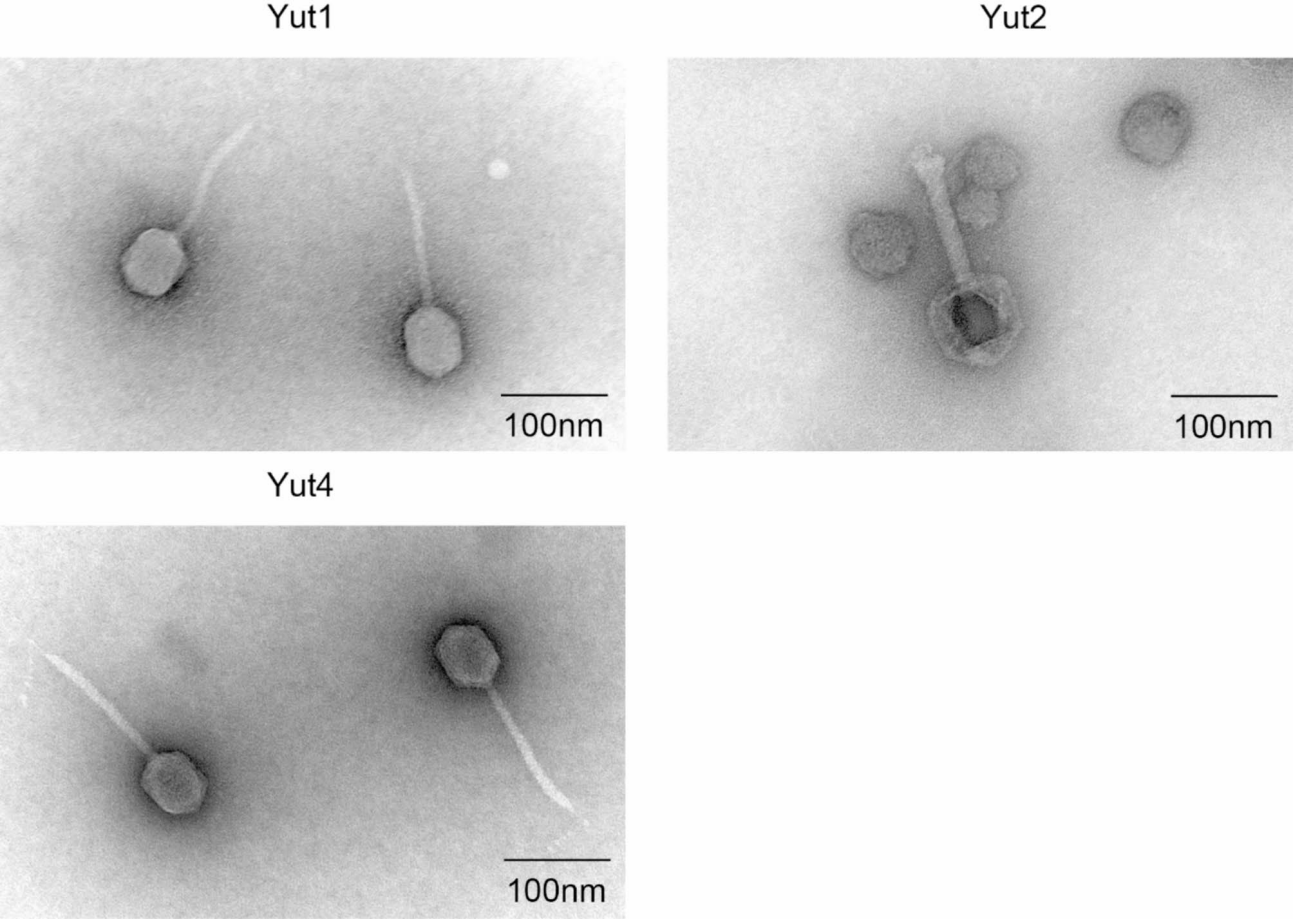
Transmission electron microscopy (TEM) images of phages Yut1, Yut2, and Yut4. Transmission electron microscope image of Yut1, Yut2, and Yut4 observed at ×50,000 magnification. Representative phages are shown.

To determine their morphological characteristics, Yut1, Yut2, and Yut4 were observed using transmission electron microscopy (TEM) (Fig. 5).

Yut1 and Yut4 appeared to belong to the *siphovirus* family, which is characterized by a long tail, with lambda phage being a representative example^16,21,26^. In contrast, Yut2 appeared to belong to the *myovirus* family, which is characterized by a relatively short, thick, and rigid tail, with T4 phage being a representative example^27^. For each phage, the head length, head width, and tail length were measured in three independent particles (Supplementary Table 5), and the average values were calculated (Table 2).

**Table 2:** Morphology of Yut1, Yut2, and Yut4.

Phage	Head (length)	Head (width)	Tail (length)
Yut1	71.2 nm	52.7 nm	139.2 nm
Yut2	101.0 nm	79.9 nm	114.7 nm
Yut4	66.6 nm	48.9 nm	149.0 nm

Yut1 exhibited a head length of 71.2 nm, head width of 52.7 nm, and tail length of 139.2 nm, indicating an elongated head and a relatively long tail. Yut4 exhibited a morphology similar to Yut1, with a head length of 66.6 nm, head width of 48.9 nm, and tail length of 149.0 nm. Although Yut1 and Yut4 exhibited highly similar morphology, filaments were observed protruding from the distal end of the tail in Yut4. Yut2 displayed a head length of 101.0 nm, head width of 79.9 nm, and tail length of 114.7 nm, featuring a relatively larger capsid and a shorter tail compared to Yut1 and Yut4.

## Discussion

In this study, we isolated a total of 34 phages against multidrug-resistant *S. maltophilia* clinical strains and evaluated their lytic spectrum by spot assay. Our initial collection of 34 phages contained a diverse set of phages as evidenced by the unique lytic activities, host ranges, and plaque morphologies. As only 31 *S. maltophilia*-specific phages are registered in the ICTV database, our collection has the potential to greatly expand the repertoire of *S. maltophilia*-specific phages. Furthermore, having used multidrug-resistant clinical strains during the isolation of our phage collection, our collection is a valuable resource for the development of phage therapy against *S. maltophilia*.

From our initial collection of 34 phages, we further characterized three phages, Yut1, Yut2, and Yut4, which demonstrated distinct infection patterns in spot assays. In liquid culture, the four phages were effective at suppressing bacterial growth at MOI’s as low as 0.01. However, while increasing the MOI resulted in faster suppression, it also accelerated the evolution of phage resistant bacteria. This accelerated emergence of resistant bacteria at high MOI is thought to be due to the strong selective pressure imposed by the higher phage concentration. These findings highlight the importance of finding optimal phage dosages that balance bacterial clearance with the emergence of phage resistant mutants. As in vitro conditions vary greatly from in vivo conditions, more work is needed to better understand phage dosages strategies, especially when used in phage cocktails.

Among the resistant mutants of 8-STEN-2 against Yut1, three isolates shared mutations in the FhuE receptor, while three resistant mutants of 23-STEN-5 against Yut4 exhibited common mutations in the colicin I receptor. Both FhuE and colicin I receptors are outer membrane proteins involved in the uptake of Fe³⁺. These findings suggest that FhuE and colicin I function as the receptors for phages Yut1 and Yut4, respectively. Supporting this, in *Escherichia coli*, FhuA, another outer membrane protein implicated in iron uptake, has been reported as the receptor for phage T5^18^. No common mutations were identified among the three resistant mutants of 15-STEN-3 against Yut2. This lack of shared mutations may reflect the presence of diverse resistance mechanisms beyond receptor mutations or the acquisition of phenotypic resistance not detectable by genomic sequencing. It is important to note that the mutated genes identified in the resistant mutants for Yut1, Yut2, and Yut4 showed no association with antibiotic uptake. Although previous studies have reported that bacteriophage-resistant mutants can concomitantly promote antibiotic resistance^28^no such effect was observed with the phages examined in this study. Therefore, the phages analyzed here are expected to have enhanced efficacy when used in combination with antibiotics.

Whole genome sequencing found that Yut1 and Yut4 possessed a genome size of approximately 60 kbp, while Yut2 had genomes of approximately 160 kbp. Phylogenetic analysis placed Yut1 and Yut4 in close proximity. Despite the high genomic similarities, Yut1 and Yut4 possessed unique host ranges, plaque morphologies, and lytic activity in liquid culture. Comparative genomic analysis revealed that although the tail proteins of Yut1 and Yut4 are generally similar, their C-terminal regions differ partially. These differences may account for the observed variation in host range between the two phages. Given the genomic similarity between the phages, further investigation may provide insights into how small genetic changes alter lysis dynamics and bactericidal activity.

None of the phages carried virulence factors, antibiotic resistance genes, lysogeny-related genes, or toxin genes. Although the relatively large genome of Yut2 did not harbor such genes, numerous hypothetical proteins with unknown functions were identified. Therefore, continuous updates of genome annotations using the latest databases are necessary to ensure accurate assessment of phage safety. Besides lacking undesirable genes, none of the phages exhibited transduction activity. These findings support the potential of these phages as promising candidates for therapeutic applications.

While our study focused on three phages (Yut1, Yut2, and Yut4), the remaining 30 phages in our collection exhibited unique lytic spectra and plaque morphologies. These findings suggest that additional novel phages may be contained within our collection. Furthermore, as the ICTV database currently only contains 31 *S. maltophilia* phages, there is incentive to add to the global database. Our study identified and characterized three phages with high potential for phage therapy. The three phages, as well as the remaining phages in our collection, are valuable tools in combating the antibiotic resistance crisis and warrant further investigation.

## Methods

### Bacterial culture conditions

*S. maltophilia* clinical isolates (8-STEN-2, 15-STEN-3, 23-STEN-5, and 25-STEN-6) were obtained from the National Center for Global Health and Medicine, Japan. Bacterial strains were streaked from − 80 °C glycerol stocks onto LB agar plates (BD Difco, Franklin Lakes, NJ, USA) and incubated overnight at 37 °C. A single colony was picked and inoculated into 2 mL of LB broth and cultured overnight at 37 °C with shaking at 200 rpm.

### Bacterial genomic DNA extraction

Strains 8-STEN-2, 15-STEN-3, 23-STEN-5, and 25-STEN-6 were cultured overnight in LB broth at 37 °C with shaking. The overnight cultures were diluted 1:10 in fresh LB broth and incubated for an additional 2 h under the same conditions. Two milliliters of each culture were then harvested, and genomic DNA was extracted using the DNeasy Blood & Tissue Kit (Qiagen GmbH, Hilden, Germany) according to the manufacturer’s protocol. Briefly, the sample was digested with proteinase K, washed with the provided buffers, and DNA was purified using spin columns. Finally, DNA was eluted with AE buffer.

### Bacterial genome sequencing, assembly

Bacterial DNA was sequenced by Nanopore long read sequencing. Bacterial DNA was prepared using the Nanopore Ligation sequencing gDNA-Native Barcoding Kit 96 V14 (SQK-NBD114.96) following the manufacturer protocol. Reads were basecalled using super-high accuracy (r1041_e82_400bps_sup_v4.3.0). Basecalled reads were concatenated using fastcat v0.22.0^29^. Nanofilt v2.8.0 was used to retain high quality reads (Q > 20)^30^. Wild type draft genomes were assembled by Flye v2.9.5 (nano-hq)^31^ and polished once by Medaka v2.0.1^32^. Polished assemblies were then annotated using Prokka v1.14.5^33^. Wild type assemblies were compared using Pyani-plus v0.0.1^33,34^.

### Sewage concentration

Sewage samples (1 L each) were collected from two sites in Tokyo. The samples were centrifuged at 6,000 × g for 15 min, and the supernatant was collected. Polyethylene glycol (PEG) 8000 and NaCl were added to final concentrations of 10% (w/v) and 4% (w/v), respectively. The mixture was stirred until fully dissolved and left at 4 °C overnight. The following day, the sample was centrifuged at 9,000 × g for 60 min. The resulting pellet was resuspended in 1 mL of saline magnesium (SM) buffer (100 mM NaCl, 50 mM Tris-HCl [pH 7.5], 7 mM MgSO_4_, and 0.01% [w/v] gelatin). The suspension was filtered through a 0.45 μm filter and stored at 4 °C.

### Phage isolation

The concentrated sewage samples (50 µL) were mixed with 100 µL of the overnight bacterial culture of each isolate in 3 mL of LB top agar (LTA; 0.5% glycerol, 0.1 M CaCl_2_) and poured onto LB agar plates (15 mL). The plates were incubated overnight at 37 °C. The following day, single plaques were picked and suspended in 100 µL of SM buffer. The phage suspension was serially diluted 10-fold up to 10^− 7^, and spot assays were performed using the corresponding host bacteria. After overnight incubation at 37 °C, single plaques were picked again and suspended in 500 µL of SM buffer. A 200 µL aliquot of the suspension was mixed with 50 µL of an overnight bacterial culture in 2 mL of LB broth and incubated for at least 2 h until the solution became clear. If the solution did not clear within 6 h, the amount of bacterial culture added was reduced, and the process was repeated. The cleared solution was filtered through a 0.45 μm filter and stored at 4 °C as the phage stock.

### Spot assay

Spot Assays were conducted using a slightly modified version of the previously described method^35^. To prepare the bacterial lawn, 300 µL of an overnight culture was added to 10 mL of LTA (0.5% agarose) and poured onto LB agar plates (30 mL). Phage suspensions were serially diluted 10-fold (from 10^0^ to 10^7^), and 2 µL of each dilution was spotted onto the bacterial lawn. After drying, the plates were incubated overnight at 37 °C to visualize plaque formation.

### Host range determination

The host range of isolated phages was tested against four clinical isolates (8-STEN-2, 15-STEN-3, 23-STEN-5, and 25-STEN-6). Each isolate was cultured overnight as described above. A 300 µL aliquot of the bacterial culture was mixed with 10 mL of LTA and poured onto LB agar plates. The isolated phage suspensions were then subjected to spot assays against all four isolates. After overnight incubation at 37 °C, plaque-forming units (PFU) were counted, and the host range was visualized in a heatmap.

### Lysis curve assay

Lysis curve assays were conducted following the established protocols^36^. Lysis curves were measured for four isolated phages: Yut1, Yut2, and Yut4. Host bacteria were cultured overnight, then diluted 100-fold with LB broth. A 180 µL aliquot of the diluted culture was added to each well of a 96-well plate. Phage solutions were prepared to achieve multiplicities of infection (MOI) of 0.01, 0.1, 1, and 10, and 20 µL of each phage solution was added to the wells. For the control, 20 µL of LB broth was added instead of phage solution. A blank control well containing 200 µL of LB broth was also prepared. Each condition was performed in triplicate (*n* = 3), and OD_600_ was measured every 10 min for 15 h. The average OD values were plotted as line graphs, and standard deviations were calculated and represented as error bars.

### Isolation and analysis of phage-resistant mutants

To isolate phage-resistant mutants, lysis curve assays were performed for 24 h at a multiplicity of infection (MOI) of 1 using each phage (Yut1, Yut2, and Yut4) against the bacterial strain from which it was originally isolated. After incubation, the cultures were streaked onto LB agar plates and incubated overnight at 37 °C. The following day, a single colony from each plate was picked and cultured overnight in LB broth. To confirm resistance, spot assays were performed using the corresponding phage. All experiments were conducted in triplicate (*n* = 3). DNA extraction and sequencing of resistant mutants was done following our previously mentioned methods. To identify mutations, Medaka v2.0.1 medaka_variant was used to identify mutations between the reference wild type and the mutants^32^.

### Phage genomic DNA extraction

To propagate phage, 20 µL of phage solution and 100 µL of overnight bacterial culture were added to 20 mL of LB broth. The mixture was incubated at 37 °C with shaking at 200 rpm until the culture cleared. If the culture did not clear within 6 h, the initial amount of bacterial culture was reduced, and the incubation was repeated. The culture was then centrifuged at 6,000 × g for 15 min, and the supernatant was filtered through a 0.45 μm filter. DNase I and RNase A were added to final concentrations of 1 U/mL and 10 µg/mL, respectively, and incubated at 37 °C for 30 min. An equal volume of polyethylene glycol (PEG) solution (10% [w/v] PEG 8000, 1 M NaCl, 5 mM Tris-HCl [pH 7.5], 5 mM MgSO_4_) was slowly added to the filtrate. The mixture was thoroughly combined by gentle inversion and left at 4 °C overnight. The following day, the samples were centrifuged at 10,000× g for 15 min at 4 °C, and the supernatant was discarded. Phage genomic DNA was then extracted from the phage pellet using the DNeasy Blood & Tissue Kit (Qiagen GmbH, Hilden, Germany), following the manufacturer’s instructions. Briefly, the sample was digested with proteinase K, washed with the provided buffers, and DNA was purified using spin columns. Finally, DNA was eluted with AE buffer.

### Phage genome sequencing and annotation

The phage genomes were extracted and submitted to AZENTA for next-generation sequencing next-generation sequencing (NGS) using the Illumina NovaSeq™ 6000 platform. Contigs were trimmed using Trimmomatic v0.39 to remove adapter sequences and low-quality reads^37^. Trimmed contigs were assembled using Unicycler v0.5.1^37,38^ and annotated by Pharokka v1.7.4^39^. All packages were run using default parameters.

### Assessment of phage-mediated transduction

To evaluate the transduction ability of phages, overnight cultures of gentamicin-resistant STEN5 were diluted 1:100 in LB broth, and 2 mL of the diluted culture was infected with phages Yut1, Yut2, or Yut4 at a multiplicity of infection (MOI) of 0.1. After 2 h of incubation at 37 °C, the cultures were filtered through 0.45 μm filters to remove bacterial cells and obtain phage solution. The phage lysates were then used to infect 2 mL cultures of gentamicin-sensitive strains STEN2 and STEN3 (diluted 1:100 from overnight cultures in LB broth) at MOI 0.1 for 40 min at 37 °C. Following infection, cultures were serially diluted to 10^− 5^ and plated on both LB agar and LB agar supplemented with gentamicin. Plates were incubated overnight at 37 °C, and colony formation was assessed the following day.

### Phylogenetic analysis

All *S. maltophilia* phage genome data was downloaded from the ICTV database and analyzed alongside the four phages sequenced in this study. A phylogenetic tree was constructed using VICTOR^16,21^. On the constructed tree, the closest ICTV-registered phages to each of the four phages were identified. The genomic similarity between each pair was assessed by calculating ANI values using the ANI Calculator^40^.

### Transmission electron microscopy (TEM)

Phage lysate (2 mL) was buffer-exchanged with 2 mL of SM buffer using an Amicon Ultra (Millipore 100 kDa), and filtered through a 0.45 μm filter. CsCl solutions were prepared at densities of 1.46 g/mL, 1.55 g/mL, and 1.63 g/mL, and were carefully layered into an ultracentrifuge tube in increasing order of density, starting with the 1.63 g/mL solution at the bottom, followed by the 1.55 g/mL solution, and then the 1.46 g/mL solution. The phage solution was gently added to the top layer. The sample was subjected to ultracentrifugation at 100,000 × g for 1 h at 4 °C using a Himac CS 100FNX Micro Ultracentrifuge (Hitachi Koki Co., Ltd., Tokyo, Japan). After centrifugation, the band located between the 1.46 g/mL and 1.55 g/mL layers was extracted transferred into a new tube. The solution was subjected to buffer exchange into SM buffer using an Amicon Ultra and filtered through a 0.45 μm filter. Copper mesh grids coated with formvar and carbon (Veco grids, Nisshin EM, Tokyo, Japan) were glow-discharged and placed onto drops of the purified sample for 1 min. The grids were rinsed with distilled water, stained with a 2% uranyl acetate solution, and examined using a transmission electron microscope (HT7700, Hitachi Ltd., Japan) at 80 kV.

## Supplementary Information

Below is the link to the electronic supplementary material.

Supplementary Material 1

## Data Availability

The genome data of *Stenotrophomonas maltophilia* strains 8-STEN-2, 15-STEN-3, 23-STEN-5, 25-STEN-6 were deposited in the GenBank under the accession numbers; AP041800-AP041804. The genome data of phages Yut1, Yut2, and Yut4 were deposited in the GenBank under the accession numbers: PV130725, PV130723, and PV130726.
