# Supplementary material for: Isolation and characterization of novel bacteriophages targeting Stenotrophomonas maltophilia
Source: Sci Rep. 2025 Aug 13;15:29743. doi: 10.1038/s41598-025-14811-5 (PMC12350841; doi:10.1038/s41598-025-14811-5)
Supplement: Supplementary file 1 — Supplementary Material 1 [file 41598_2025_14811_MOESM1_ESM.docx]

**Supplementary information**

**Supplementary Figures**


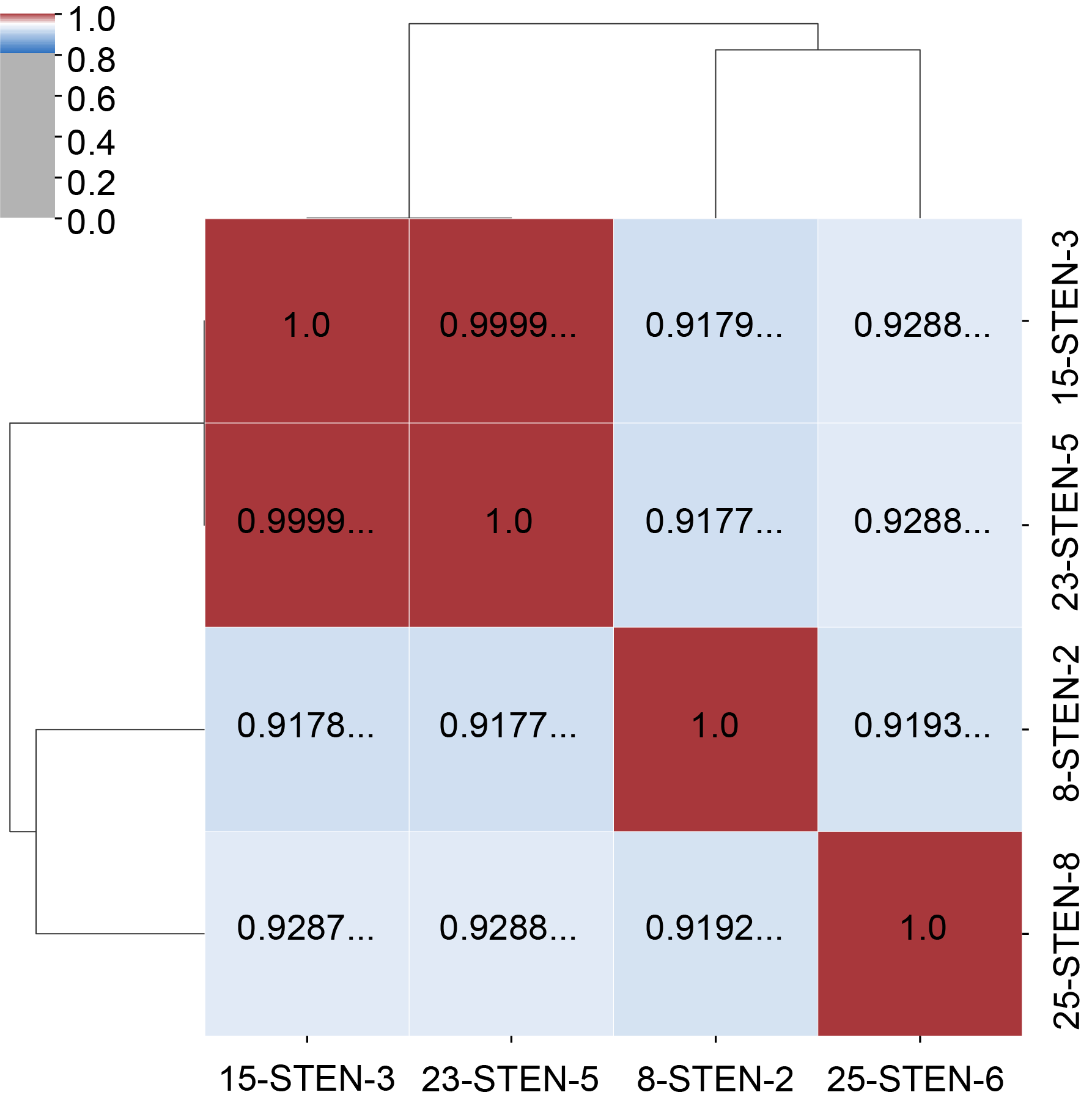


**Supplementary Figure 1** Comparison of whole genome sequences and phylogenetic tree constructed based on the sequence alignment from four *S. maltophilia* clinical isolates using pyANI-plus.

.


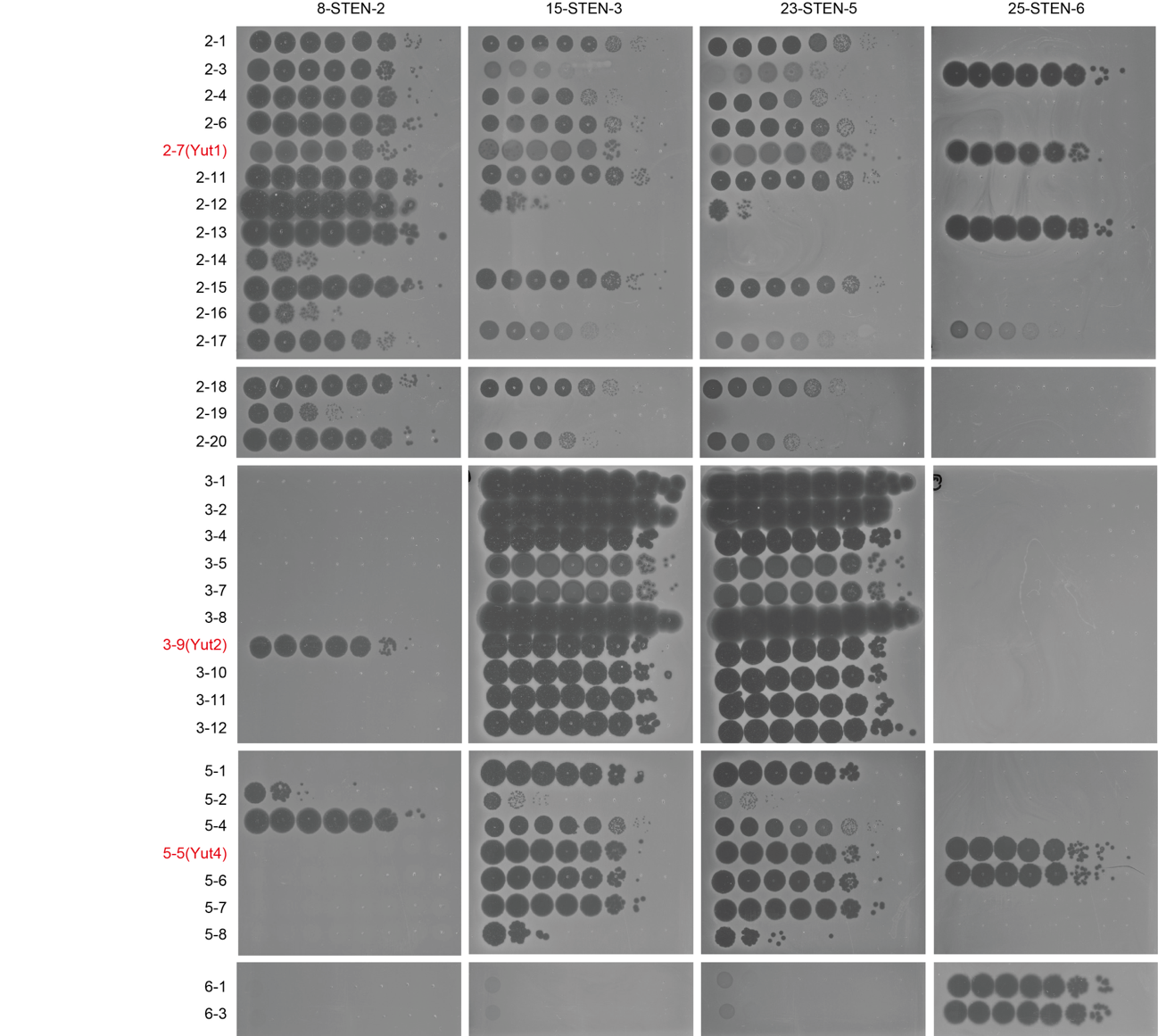


**Supplementary Figure 2** Spot test results of 34 phages isolated in this study on four *S. maltophilia* clinical isolates.


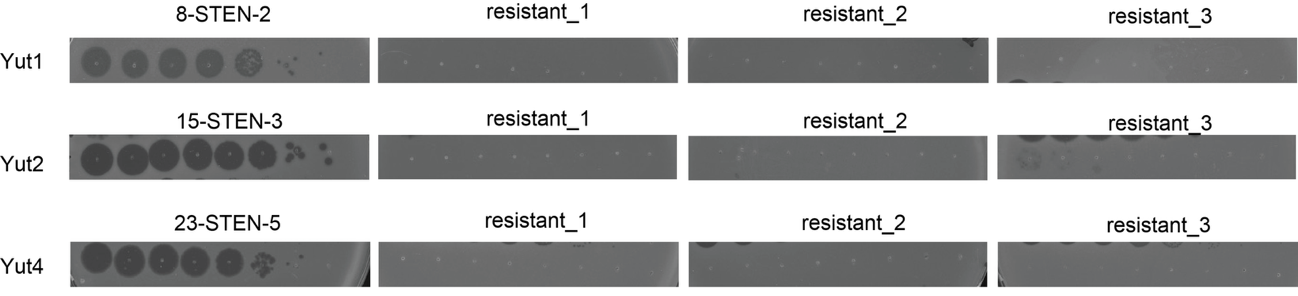


**Supplementary Figure 3** Spot test results of phages against resistant mutants. Three resistant mutant strains were isolated for each phage-host combination: 8-STEN-2 against Yut1, 15-STEN-4 against Yut2, and 23-STEN-5 against Yut4.


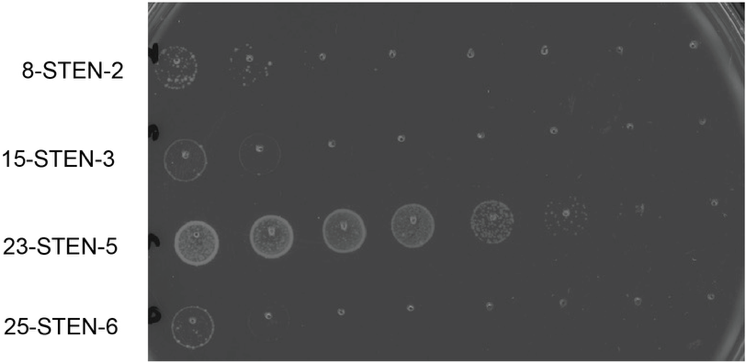


**Supplementary Figure 4** Spot test results of the four *S. maltophilia* clinical isolates on gentamicin LB agar plates.

**
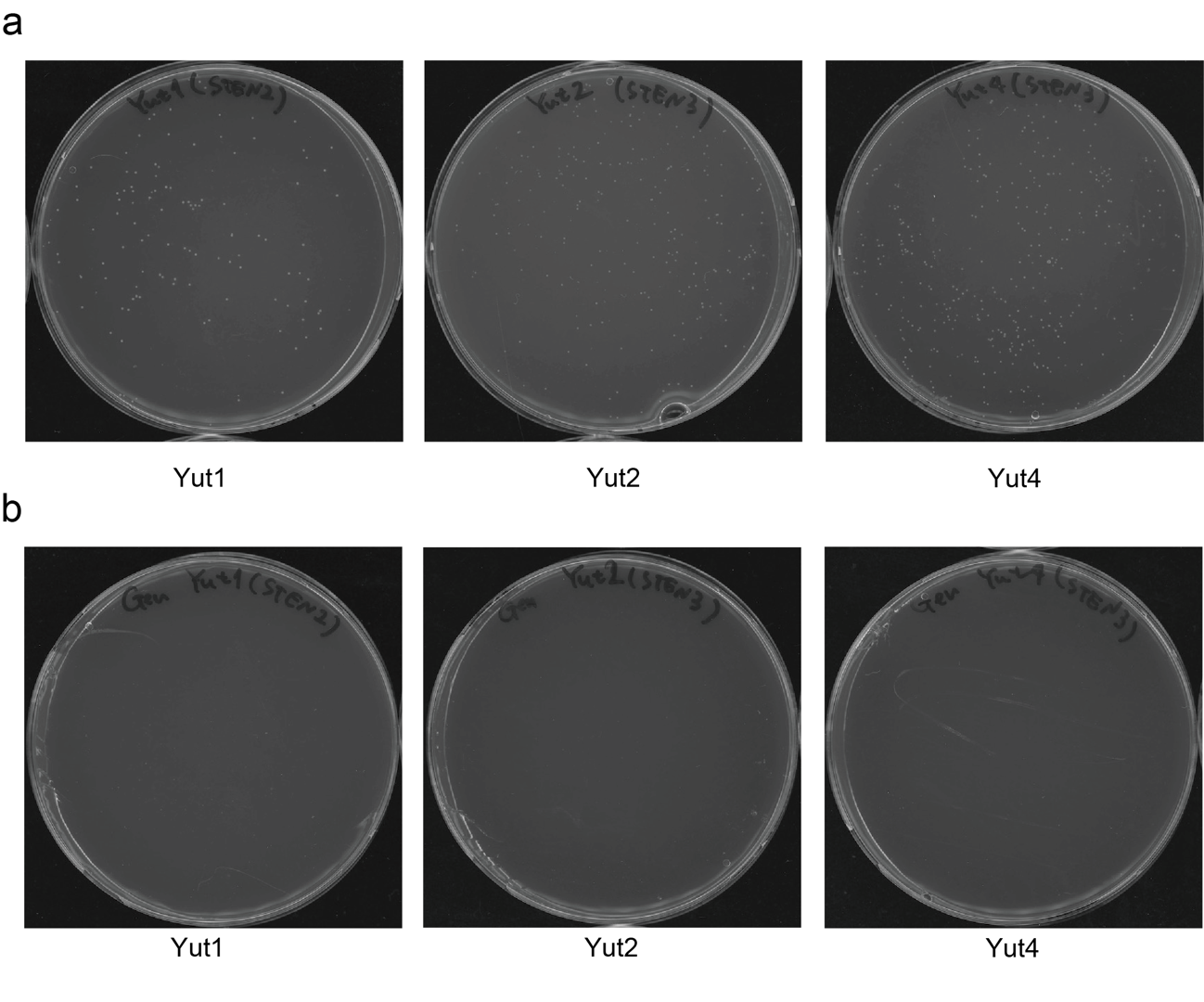
**

**Supplementary Figure 5.** Evaluation of transduction capability by phages Yut1, Yut2, and Yut4. To assess whether Yut1, Yut2, and Yut4 are capable of mediating transduction, each phage was propagated on a gentamicin-resistant donor strain (STEN5) and subsequently used to infect gentamicin-sensitive recipient strains (STEN2 for Yut1, STEN3 for Yut2 and Yut4). (a) Growth of recipient strains on LB. (b) Growth of recipient strains on gentamicin.


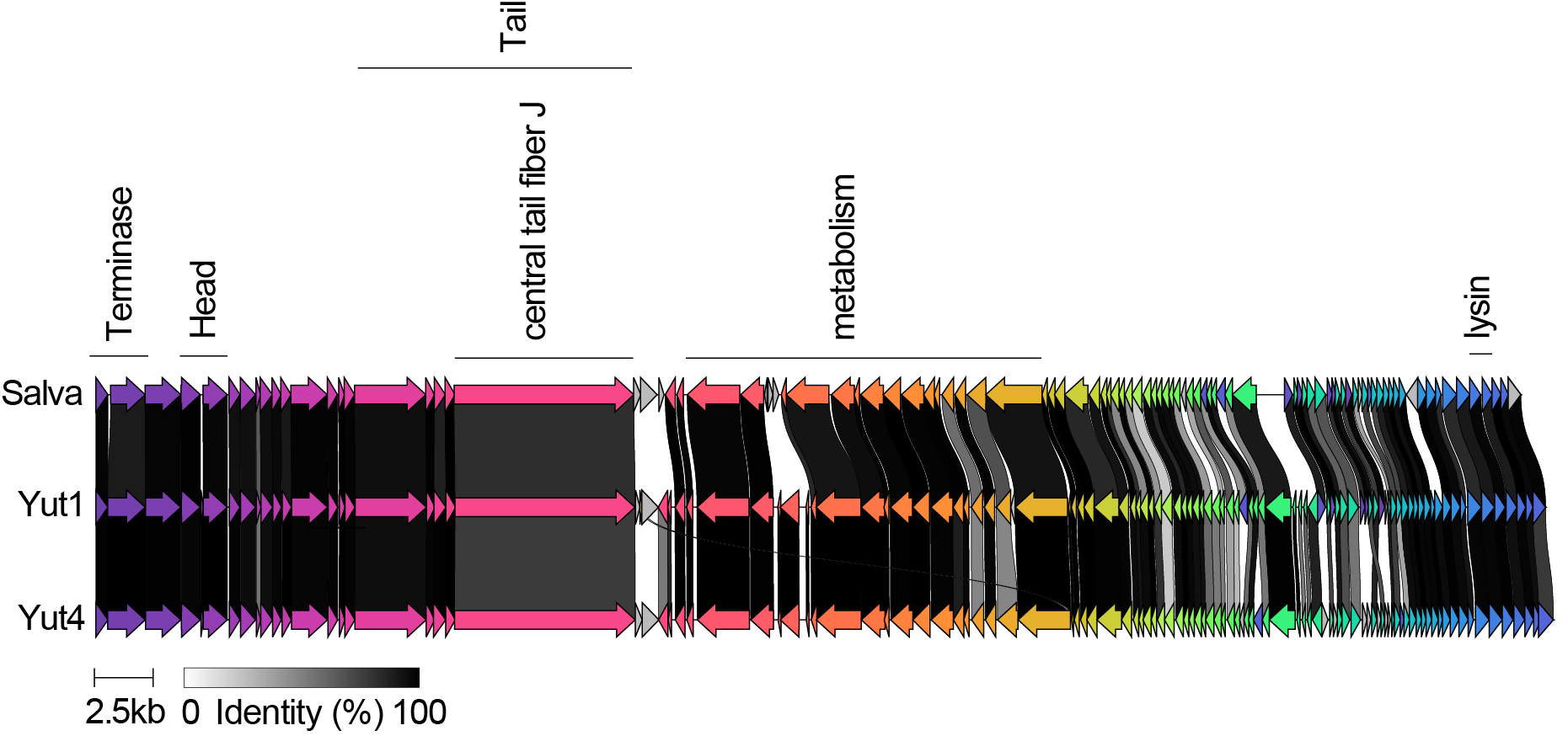


**Supplementary Figure 6.** Genome alignment of Yut1, Yut4, and Salva phages using Clinker.


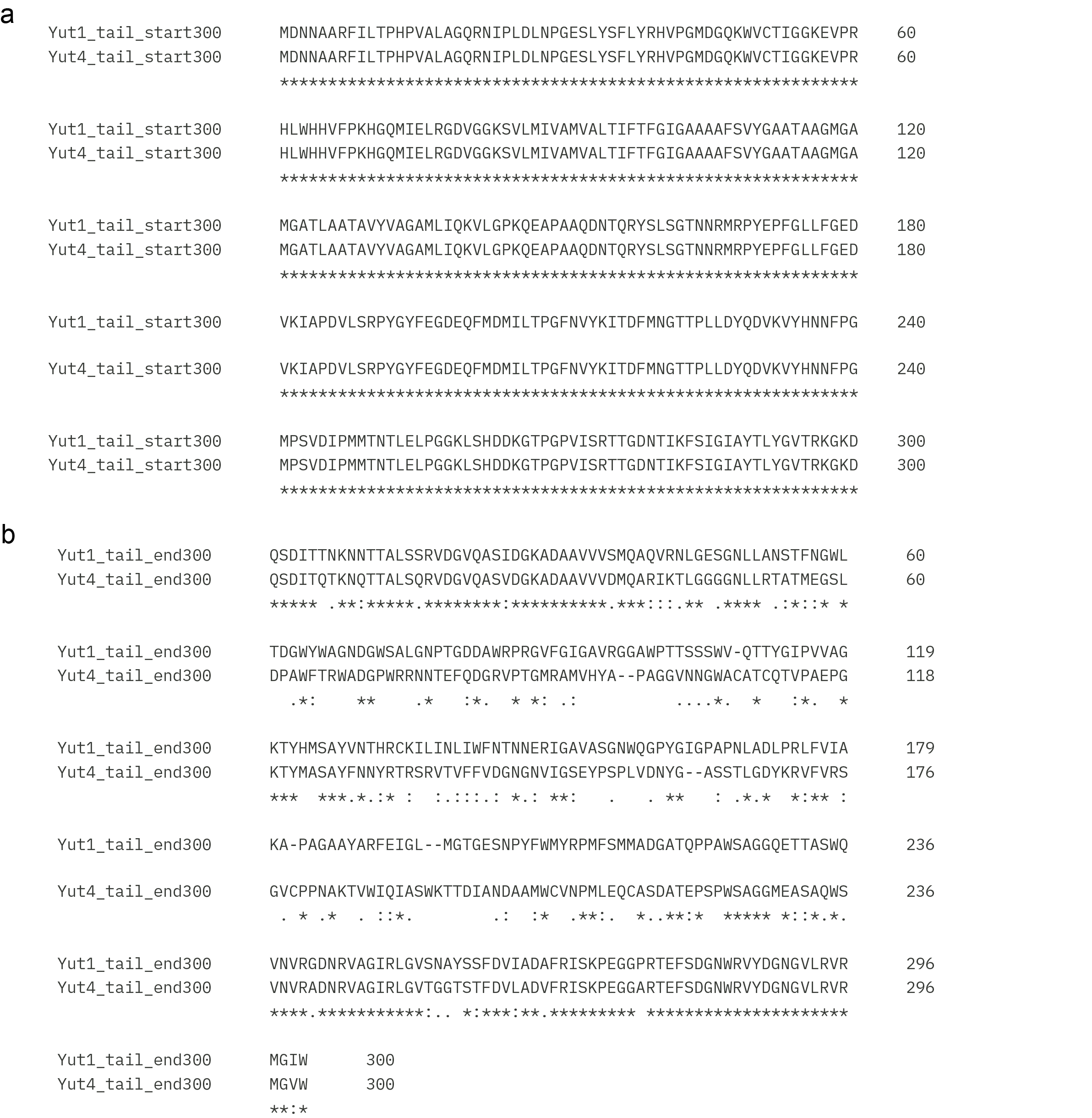


**Supplementary Figure 7.** Sequence alignment of the central tail fiber protein J of Yut1 and Yut4 using ClustalOmega[^24^](https://paperpile.com/c/eIJBWe/XZQiM)^,^[^25^](https://paperpile.com/c/eIJBWe/yQHUn). (a) Alignment of the N-terminal 300 amino acids. (b) Alignment of the C-terminal 300 amino acids.

**Supplementary Tables**

**Supplementary Table 1 Infection Profiles of Bacterial Strains Used for Phage Isolation**

| Bacterial name | Co-infecting Bacteria | Antibiotic used |
| --- | --- | --- |
| 8-STEN-2 | *-* | **Teicoplanin + Levofloxacin** |
| 15-STEN-3 | *Candida species* | **Levofloxacin + Isoniazid + Tmp-Smx + Meropenem + Micafungin + Vancomycin + Voriconazole** |
| 23-STEN-5 | *Pseudomonas aeruginosa* | **Meropenem + Micafungin + Minocycline** |
| 25-STEN-6 | *Pseudomonas aeruginosa,*  *Escherichia coli,*  *Klebsiella pneumoniae* | **Piperacillin/Tazobactam + Minocycline + Levofloxacin + Tmp-Smx + Micafungin** |

**Supplementary Table 2 Antibiotic Susceptibility of *S. maltophilia***

| Bacterial name | Drug name | MIC (μg/mL) | CLSI* |
| --- | --- | --- | --- |
| 8-STEN-2 | Ceftazidime | 4 | S |
|  | Minocycline | ≦1 | S |
|  | Levofloxacin | ≦0.5 | S |
|  | Tmp-smx | ≦1 | S |
| 15-STEN-3 | Ceftazidime | ≧32 | R |
|  | Minocycline | ≦1 | S |
|  | Levofloxacin | 8 | R |
|  | Tmp-smx | ≦1 | S |
| 23-STEN-5 | Ceftazidime | ≧32 | R |
|  | Minocycline | ≦1 | S |
|  | Levofloxacin | ≦0.5 | S |
|  | Tmp-smx | ≦1 | S |
| 25-STEN-6 | Ceftazidime | ≧32 | R |
|  | Minocycline | - | - |
|  | Levofloxacin | 4 | I |
|  | Tmp-smx | - | - |

*The abbreviation of Clinical and Laboratory Standards Institute. S stands for Susceptible, I for Intermediate, and R for Resistant. A dash (-) indicates that the test was not performed.

**Supplementary Table 3 Phage Isolation Hosts**

| Phage | Host Strain |
| --- | --- |
| Φ2-1 | 8-STEN-2 |
| Φ2-3 | 8-STEN-2 |
| Φ2-4 | 8-STEN-2 |
| Φ2-6 | 8-STEN-2 |
| Φ2-7(Yut1) | 8-STEN-2 |
| Φ2-8 | 8-STEN-2 |
| Φ2-11 | 8-STEN-2 |
| Φ2-12 | 8-STEN-2 |
| Φ2-13 | 8-STEN-2 |
| Φ2-14 | 8-STEN-2 |
| Φ2-15 | 8-STEN-2 |
| Φ2-16 | 8-STEN-2 |
| Φ2-17 | 8-STEN-2 |
| Φ2-18 | 8-STEN-2 |
| Φ2-19 | 8-STEN-2 |
| Φ2-20 | 8-STEN-2 |
| Φ3-1 | 15-STEN-3 |
| Φ3-2 | 15-STEN-3 |
| Φ3-4 | 15-STEN-3 |
| Φ3-5 | 15-STEN-3 |
| Φ3-6 | 15-STEN-3 |
| Φ3-7 | 15-STEN-3 |
| Φ3-8 | 15-STEN-3 |
| Φ3-9(Yut2) | 15-STEN-3 |
| Φ3-10 | 15-STEN-3 |
| Φ3-11 | 15-STEN-3 |
| Φ3-12 | 15-STEN-3 |
| Φ5-1 | 23-STEN-5 |
| Φ5-2 | 23-STEN-5 |
| Φ5-4 | 23-STEN-5 |
| Φ5-5(Yut4) | 23-STEN-5 |
| Φ5-6 | 23-STEN-5 |
| Φ5-7 | 23-STEN-5 |
| Φ6-1 | 25-STEN-6 |
| Φ6-3 | 25-STEN-6 |

**Supplementary Table 4 Mutated genes identified in phage-resistant bacterial isolates**

**Supplementary Table 4-1 8-STEN-2 mutants resistant to Yut1**

| bacteria | position | mutated gene | Reference | Mutation | Qscore |
| --- | --- | --- | --- | --- | --- |
| resistant① | 1817425 | hypothetical protein | TG | T | 9.403 |
|  | 2040930 | non-coding region | G | GC | 9.456 |
|  | 2041073 |  | A | AG | 16.484 |
|  | 3600992 | **FhuE receptor** | G | C | 69.25 |
| resistant② | 2040930 | non-coding region | G | GC | 14.911 |
|  | 2041073 |  | A | AG | 8.57 |
|  | 3600890 | **FhuE receptor** | AGCCAGAACGAGTTCGC | A | 407.07 |
| resistant③ | 1817425 | hypothetical protein | TG | T | 8.864 |
|  | 1859516 | non-coding region | GT | G | 5.532 |
|  | 2039694 | non-coding region | G | GC | 6.975 |
|  | 2040930 |  | G | GCC | 23.22 |
|  | 3601013 | **FhuE receptor** | A | T | 69.25 |

**Supplementary Table 4-2 15-STEN-3 mutants resistant to Yut2**

| bacteria | position | mutated gene | Reference | Mutation | Qscore |
| --- | --- | --- | --- | --- | --- |
| resistant① | 3754594 | hypothetical protein | G | A | 69.25 |
| resistant② | 1157269 | Twitching mobility protein | ACCGCAGCGTG | A | 190.636 |
|  | 1503059 | non-coding region | CG | C | 8.31 |
|  | 2065334 | non-coding region | A | AG | 6.111 |
| resistant③ | 398463 | hypothetical protein | G | A | 69.25 |
|  | 1503059 | non-coding region | CG | C | 10.71 |
|  | 3132225 | UDP-glucose 6-dehydrogenase TuaD | C | CA | 69.25 |

**Supplementary Table 4-3 23-STEN-5 mutants resistant to Yut4**

| bacteria | position | mutated gene | Reference | Mutation | | Qscore |
| --- | --- | --- | --- | --- | --- | --- |
| resistant① | 381096 | Adenosylhomocysteinase | C | CT | 33.092 | |
|  | 754053 | **Colicin I receptor** | AGG | A | 18.125 | |
|  | 1763195 | Single-stranded-DNA-specific exonuclease RecJ | GTA | G | 12.192 | |
|  | 1763199 |  | C | A | 12.22 | |
|  | 1763208 |  | G | C | 9.173 | |
|  | 1763396 |  | G | A | 69.25 | |
|  | 1763398 |  | T | C | 33.094 | |
|  | 1764905 | non-coding region | A | G | 7.195 | |
|  | 2958519 | hypothetical protein | G | A | 69.25 | |
|  | 3181039 | Protein QmcA | T | C | 8.463 | |
|  | 3404750 | Formate dehydrogenase-O major subunit | A | G | 33.094 | |
|  | 3404759 |  | T | C | 15.55 | |
|  | 3404767 |  | C | G | 5.013 | |
| resistant② | 754053 | **Colicin I receptor** | AGG | A | 28.883 | |
|  | 1050500 | non-coding region | CG | C | 7.23 | |
|  | 2958519 | hypothetical protein | G | A | 69.25 | |
| resistant③ | 752462 | **Colicin I receptor** | GC | G | 28.34 | |

**Supplementary Table 5 Raw Morphology Data of Yut1, Yut2 and Yut4.**

| Phage | Head(length) | Head(width) | Tail(length) |
| --- | --- | --- | --- |
| Yut1 | 71.3, 72.8, 69.6 nm | 51.1, 51.5, 55.4 nm | 140.5, 144.0, 133.0 nm |
| Yut2 | 93.9, 108, 101 nm | 87.3, 71.3, 81.1 nm | 108.0, 122.0, 114.0 nm |
| Yut4 | 67.9, 64.7, 67.2 nm | 46.5, 51.2, 48.9 nm | 154.0, 141.0, 151.0 nm |
